# New Appliances and Things Medical

**Published:** 1897-11-13

**Authors:** 


### NEW APPLIANCES AND THINGS MEDICAL.

[We shall be glad to receive, at our Office, 28 & 29, Southampton Street, Strand, London, W.C., from the manufacturers, specimens of all new preparations and appliances which may be brought out from time to time.]

### DIGESTENZYMES (DAMANCY).

(Agents: Arthur and Co., 69, Berners Street.)

These tablets, which are manufactured by the Damancy Drug Company, have chocolate as their agreeable basis, and incorporated with this basis are the ferments, diastase, pepsin, and pancreatin. Where, as is often the case, it is desirable to assist the natural processes of digestion, the administration of these tablets at the commencement of a meal is likely to be followed by the best of results, because the combination of the above-mentioned ferments ensures that when swallowed digestion commences at once and is continued throughout the alimentary tract, whether the reaction be acid or alkaline. We feel justified in recommending this new preparation to the notice of the profession.

### MEADOW SWEET CHEESE.

(Meadow Sweet Cheese Co., 65, Duke Street, Liverpool.)

This cheese, of which we have received samples, is of excellent quality; it is particularly rich in cream and of most agreeable flavour. As a food it is highly economical both in a physiological and in a pecuniary sense, that is to say, it can be digested and absorbed by the system without loss or waste, and it can be purchased at a price which compares favourably not only with other varieties of cheese, but with other varieties of food. Samples of the cheese are securely and carefully packed in tinfoil and enclosed in cardboard cases. The packets can be easily opened, and by selecting a size appropriate to one's needs one can ensure always having a perfectly fresh cheese at a low price in the best of condition.

### CONDENSED MILK.

(Agents: Messrs. Edwin Fischel and Co., 11, Rood Lane, E.C.; and Messrs. Joseph Travers and Sons, Limited, 119, Cannon Street, E.C.)

The Bernese Alps Milk Company, through their London representatives, Messrs. Edwin Fischel and Co., has sent us a sample of the "Sledge Brand" of sterilised and unsweetened condensed milk. The sample received was of a rich cream colour, and contained according to an analysis 35·5 per cent. of dry milk solids, of which 9·5 per cent. was milk fat. It contained no sugar or other preservative. Mixed in the proportion of one part of the condensed milk to two parts of water it made a milk equal in quality and flavour to a milk of great purity. In all respects, therefore, the "Sledge Brand" of unsweetened condensed milk as submitted to us is of excellent quality.

### PACKARD'S LACTEOLA.

(A. J. White, Limited, 43, Farringdon Road, London.)

This preparation is undoubtedly a food of some value, the fat exists in a state of fine emulsion, and the carbohydrate constituent in such a form that it can be absorbed from the alimentary tract almost as soon as it reaches it. For the debilitated or consumptive Lacteola appears to us to be a preparation which should encourage the putting on of weight, and improve the general nutrition. Though somewhat unappetising in appearance, a trial of it will prove that it is by no means disagreeable or unpalatable.

### ICHTHYOL AND GLYCERINE SUPPOSITORIES FOR VAGINAL USE.

(Wyleys, Limited, Coventry and Birmingham.)

Dr. Leslie Callaghan has introduced to our notice these ichthyol suppositories, which he has had made for him for use in gynæcological work. Ichthyol has for some time been much employed as a local application, being generally introduced by means of a tampon. There are, however, many objections to this method, one of which is the unpleasantness sometimes caused by air being driven into the vagina when the tampon is inserted through a speculum. These glycerine suppositories entirely overcome this difficulty, and make it quite possible for the patient herself to make the application in the evening, a matter of no small importance in view of the desirability of rest in the horizontal position being maintained when it is wished to keep any medication in contact with the os uteri. The suppositories are stated to contain 10 per cent. of ichthyol.

### GLANDULEN.

(Thomas Christy and Co., 25, Lime Street, London, E.C.)

This is an extract prepared from the bronchial glands of healthy and freshly-killed sheep. Following on the lines of other well-known extracts of glands, it is suggested that this new preparation may be of material use in the treatment of certain diseases of the respiratory system. More fanciful theories have been followed by practical results, we therefore commend this new preparation to the notice of the profession. The dose for an adult is one tablet three times a day, and gradually increased in number up to 15 per diem.

